# Unveiling the promise: Exosomes as game‐changers in anti‐infective therapy

**DOI:** 10.1002/EXP.20230139

**Published:** 2024-03-12

**Authors:** Vivek P. Chavda, Guanghong Luo, Rajashri Bezbaruah, Tutumoni Kalita, Anupam Sarma, Gitima Deka, Yanhong Duo, Bhrigu Kumar Das, Yesha Shah, Humzah Postwala

**Affiliations:** ^1^ Department of Pharmaceutics and Pharmaceutical Technology L. M. College of Pharmacy Ahmedabad Gujarat India; ^2^ Department of Radiation Oncology Shenzhen People's Hospital (The Second Clinical Medical College, Jinan University, The First Affiliated Hospital, Southern University of Science and Technology) Shenzhen Guangdong China; ^3^ Department of Pharmaceutical Sciences Faculty of Science and Engineering Dibrugarh University Dibrugarh Assam India; ^4^ School of Pharmaceutical Sciences Girijananda Chowdhury University, Azara Guwahati Assam India; ^5^ College of Pharmacy Yeungnam University Gyeonsan Republic of Korea; ^6^ Wyss Institute for Biologically Inspired Engineering Harvard University Boston Massachusetts USA; ^7^ PharmD Section L. M. College of Pharmacy Ahmedabad Gujarat India

**Keywords:** antibacterial, anti‐infective, antiviral, exosomes, extracellular vesicles, micro vesicles, nano carrier, viral infection

## Abstract

Extracellular vesicles (EVs)‐based intercellular communication (through exosomes, microvesicles, and apoptotic bodies) is conserved across all kingdoms of life. In recent years, exosomes have gained much attention for targeted pharmaceutical administration due to their unique features, nanoscale size, and capacity to significantly contribute to cellular communication. As drug delivery vehicles, exosomes have several advantages over alternative nanoparticulate drug delivery technologies. A key advantage lies in their comparable makeup to the body's cells, which makes them non‐immunogenic. However, exosomes vesicles face several challenges, including a lack of an effective and standard production technique, decreased drug loading capacity, limited characterization techniques, and underdeveloped isolation and purification procedures. Exosomes are well known for their long‐term safety and natural ability to transport intercellular nucleic acids and medicinal compounds across the blood‐brain‐barrier (BBB). Therefore, in addition to revealing new insights into exosomes’ distinctiveness, the growing availability of new analytical tools may drive the development of next‐generation synthetic systems. Herein, light is shed on exosomes as drug delivery vehicles in anti‐infective therapy by reviewing the literature on primary articles published between 2002 and 2023. Additionally, the benefits and limitations of employing exosomes as vehicles for therapeutic drug delivery are also discussed.

## Graphical abstract

A comprehensive review to reveal new insights into exosomes’ distinctiveness, the growing availability of new analytical tools that drive the development of next‐generation synthetic systems were presented. Herein, light is shed on exosomes as drug delivery vehicles in anti‐infective therapy. Additionally, the benefits and limitations of employing exosomes as vehicles for therapeutic drug delivery are also discussed.

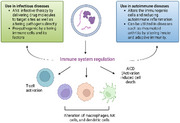

## 1 INTRODUCTION

Intercellular communication is crucial for maintaining homeostasis in multicellular systems, and the down‐regulation of these communication pathways has been directly linked to the pathogenesis and advancement of infections.^[^ ^1^ ^]^ Chemical messengers, mainly in the form of extracellular vesicles (EVs), have been discovered to facilitate intercellular communication.^[^ ^2^ ^]^ EVs, membranous vesicles released by cells into the extracellular space, facilitate the selective transportation of macro‐ and micro‐nutrients, as well as nucleic acids, which may be absorbed by nearby or distant recipient cells, where they subsequently initiate numerous biological functions by modulating the phenotype the recipient cells.^[^ ^3^ ^]^ Based on their origin and size, EVs can be categorized into three different categories: exosomes (30−150 nm in diameter), microvesicles (50 nm to 1 µm in diameter), and apoptotic bodies (50 nm to 5 µm in diameter).^[^ ^4^ ^]^ Due to their unique features, nanoscale dimensions, and potential to significantly contribute to cellular communication, exosomes have particularly been regarded as one of the most intriguing molecules for targeted drug delivery. Consequently, they have received considerable attention in recent years.^[^ ^5^ ^]^

In 1983, Harding et al. and Pan et al. demonstrated the production of microscopic membranous vesicles in the supernatant of sheep reticulocytes, leading to the discovery of exosomes.^[^ ^6^, ^7^ ^]^ While developing red blood cells, these researchers observed the formation of “an intracellular sac filled with the small membrane‐enclosed structure of nearly uniform size”. In contrast to the ingestion of exogenous molecules into the cell during endocytosis, these newly discovered entities formed intracellular vesicles and discharged contents into the extracellular space. Hence, in 1987, Johnstone et al. introduced the term ‘exosomes’ to describe these vesicles.^[^ ^8^ ^]^ However, a similar terminology, ‘exosome complex’ had previously been used by Trams et al. in 1981 to describe protein‐bound intracellular complexes associated with the degradation of nucleic acids.^[^ ^9^ ^]^ Furthermore, Wang et al. observed a considerable rise in worldwide exosome research publications from 1994 to 2017, implying that exosome research has been an intriguing and fast‐growing area of study.^[^ ^10^ ^]^

**FIGURE 1:**
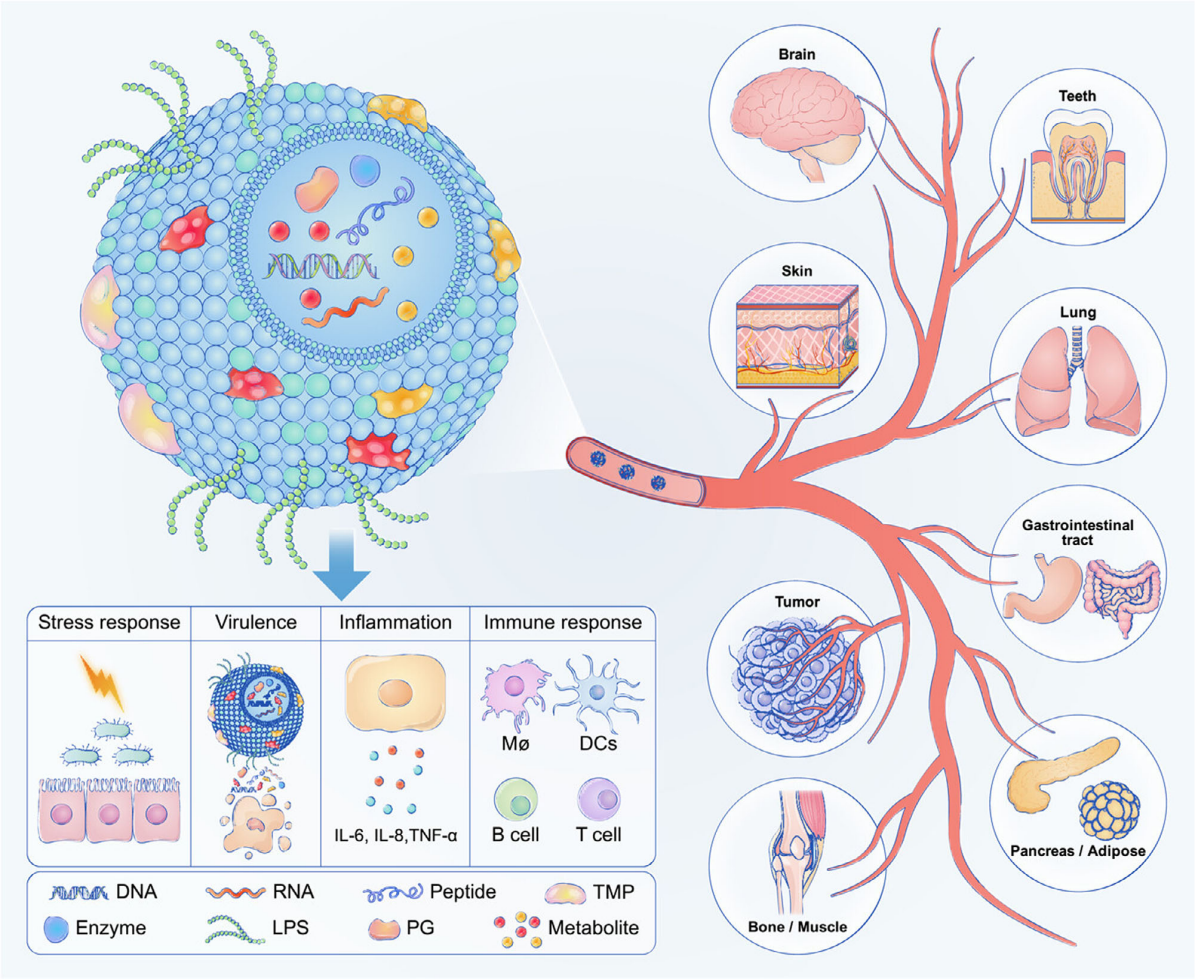
Diagrammatic representation of exosomes composition. Exosomes are small vesicles, with a diameter ranging from 30 to 100 nm that are released by different types of cells. They consist of a lipid bilayer membrane that encloses a diverse range of biomolecules, such as proteins, lipids, and nucleic acids. These biomolecules have essential functions in transmitting information between cells and can regulate a wide range of cellular activities. The composition of exosomes is contingent upon their cellular source and physiological circumstances. Essential constituents of exosomes comprise: Transmembrane proteins are proteins that extend over the exosome membrane and play a role in the formation, movement, and contact with targeted cells of exosomes. Cytosolic proteins are found inside the exosome lumen and can perform several tasks, including as enzymatic activity, signaling, and immunological regulation. Lipids form the membrane of exosomes and play a role in maintaining its stability and fluidity. Nucleic acids found in exosomes encompass a diverse range, comprising mRNA, miRNA, and pieces of DNA. The transfer of these nucleic acids to recipient cells can exert an impact on their gene expression. Abbreviations: DC, dendritic cell; IL, interlekin; LPS, lipopolysaccharide; PG, peptidoglycan; TMP, transmembrane protein; TNF, tumor necrosis factor. Adapted under the terms of CC‐BY license.^[^ ^24^ ^]^ Copyright 2023, The Authors, published by John Wiley & Sons Australia, Ltd on behalf of iMeta Science.

Exosomes are membranous lipid bilayer structures comprising abundant biomolecules like lipids, proteins, carbohydrates, nucleic acids, and so on.^[^ ^11^ ^]^ These vesicles originate from endosomes. However, the composition of exosomes may vary depending on various factors, including origin and release, and the physiological and pathological condition of the parent endocytic cell. Several exosome databases and compendiums, including ExoCarta, The Urinary Exosome Protein Database, EV‐TRACK, MiRandola 2017, Vesiclepedia, ExorBase, EVmiRNA, ExoBCD, and so on, have been curated to provide information about the molecular contents of exosomes.^[^ ^12^, ^13^ ^]^ The most widely used database (ExoCarta), has so far reported 9769 proteins, 2838 miRNAs, and 3408 mRNAs to be present in biological samples investigated. Most exosomes, regardless of origin, contain a common set of proteins. These exosome‐associated proteins include: adhesion proteins (integrins), heat shock proteins (HSPs, such as HSP70 and HSP90), tetraspanins (CD63, CD9, CD82, and CD81), cytoskeletal proteins (actin), major histocompatibility complex (MHC) proteins (MHC I and II), and fusion proteins (GTPases, flotillin, and annexins).^[^ ^14^ ^]^ Some of these proteins are abundantly present in exosomes and function as biomarkers for detecting infectious agents. On the other hand, endocytic proteins such as CD55 and CD59, Alix, thrombospondin, lactadherin, and Tumor Susceptibility Gene 101 (TSG 101) act as cargoes for cellular communication. In addition to proteins, exosomes contain abundant lipids. Several lipids (phosphatidylinositol, sphingomyelin, phosphatidylcholine, glycosphingolipids, phosphatidylserine, cholesterol, ceramide, saturated fatty‐acid chains, and phosphatidylethanolamine) make up the exosome lipid bilayer. These lipids provide rigidity to the exosome membrane and play critical roles in exosome formation and efflux of vesicular biomolecules when fused with the plasma membrane.^[^ ^14^, ^15^, ^16^ ^]^ The composition of exosomes is briefly highlighted and diagrammatically represented in Table 1 and Figure 1, respectively.

**TABLE 1:** Composition of exosomes.

Biomolecule	Types
Proteins^[^ ^17^, ^18^, ^19^ ^]^	Integrins (adhesion proteins), major histocompatibility complex (MHC) proteins (MHC I and II), heat shock proteins (HSPs, such as HSP70 and HSP90), tetraspanins (CD63, CD9, CD82, and CD81), fusion proteins (GTPases, flotillin, and annexins), cytoskeletal proteins (actin), proteins of endocytic origin (CD55 and CD59), and other proteins (Alix, Thrombospondin, Lactadherin, and TSG 101)
Lipids^[^ ^20^, ^21^ ^]^	Phosphatidylinositol, sphingomyelin, phosphatidylcholine, glycosphingolipids, phosphatidylserine, cholesterol, ceramide, saturated fatty‐acid chains, and phosphatidylethanolamine
Nucleic acids^[^ ^22^, ^23^ ^]^	DNA, RNA and miRNA

## 2 ROLE OF ENDOSOMAL SORTING COMPLEX REQUIRED FOR TRANSPORT (ESCRT) COMPLEX IN EXOSOMES FORMATION, RELEASE, AND UPTAKE

The standard process of exosomes formation entail three major steps: synthesis of endocytic vesicles from the cellular membrane, development of multivesicular bodies (MVBs) induced by internalized vesicle membrane budding, and efflux of vesicular contents.^[^ ^25^ ^]^ Early endosomes are initially produced from endocytic vesicles formed due to plasma membrane invagination. Biologically active molecules accumulate inside these endosomes, and late endosomes are formed as they mature. The MVBs are formed when the limiting membrane of late endosomes is indented, and they contain many sub‐cellular structures (ILVs) inside the lumen.^[^ ^16^, ^26^, ^27^, ^28^, ^29^ ^]^ Either the endosomal sorting complexes required for transport (ESCRT) pathway or the non‐ESCRT pathway can facilitate the MVBs biogenesis. After the production of these MVBs, two possible consequences can potentially follow.^[^ ^30^ ^]^ The MVBs can either fuse with lysosomes and destroy the ILVs, or allow ILVs to exit as exosomes by integrating with the outer membrane. Following their secretion, exosomes may persist in the extracellular environment or move actively in body fluids. Furthermore, following their absorption by nearby and distant cells, exosomes can regulate the behaviors of target cells.^[^ ^25^, ^26^ ^]^

As supported by several studies, the formation of the ESCRT complex is required for exosome biogenesis. The ESCRT‐0 subunit, hepatocyte growth factor‐regulated tyrosine kinase substrate (HRS), is particularly critical for exosome biogenesis. Different types of cell lines, including human embryonic kidney cell line 293 (HEK 293), mouse dendritic cells, Henrietta Lacks (HeLa) cancer cells, and squamous carcinoma cells, experience reduced exosome secretion because of HRS depletion. Immortalized retinal pigment epithelial cells 1 (RPE 1) and tumor cells also experience decreased exosomal secretion due to TSG 101 suppression.^[^ ^31^, ^32^, ^33^, ^34^ ^]^

The ESCRT complex is a group of four proteins (ESCRT‐0, I, II, and III) and other related proteins (Vacuolar Protein Sorting‐Associated Protein 4 (VPS 4), TSG 101, Charged Multivesicular Body Protein 4 (CHMP 4), and Alix) that work together to facilitate the overall exosome formation process.^[^ ^13^ ^]^ The process is initiated with the assistance of ESCRT‐0 ubiquitin‐binding subunits, which distribute ubiquitinated proteins to particular sections of the endosome membrane. In conjunction with ESCRT‐II, TSG 101 of ESCRT‐I, which ESCRT‐III further induces membrane invagination. Consequently, ILVs are formed following a VPS 4‐powered cleaving of the ESCRT complex from the MVB membrane.^[^ ^27^, ^28^, ^29^ ^]^ Several studies have correlated exosome proteins related to ESCRT proteins (such as TSG 101 and CHMP 4) with membrane budding and efflux of vesicular contents.^[^ ^35^, ^36^ ^]^ Furthermore, ESCRT proteins have been reported to exhibit exosomal cargo selection, which is facilitated by the association with syndecan.^[^ ^37^ ^]^

However, previous studies have also implied the existence of an ESCRT‐independent pathway by demonstrating that ESCRT inhibition does not affect exosome formation and release.^[^ ^38^, ^39^ ^]^ Both macromolecules and micromolecules, including lipids, cholesterol, and proteins (particularly tetraspanins, ceramide, phosphatidic acid, heat shock proteins, and so on), have been demonstrated to actively engage in the non‐ESCRT pathway. For example, by communicating with certain receptors present in the plasma membrane, tetraspanin‐enriched microdomains (TEMs) formed by tetraspanins can expedite exosomes biogenesis, binding, uptake, release, and cargo selection.^[^ ^16^, ^40^ ^]^ Additionally, it has been reported that sphingolipids such as ceramides stimulate membrane budding to generate vesicles.^[^ ^41^ ^]^

**FIGURE 2:**
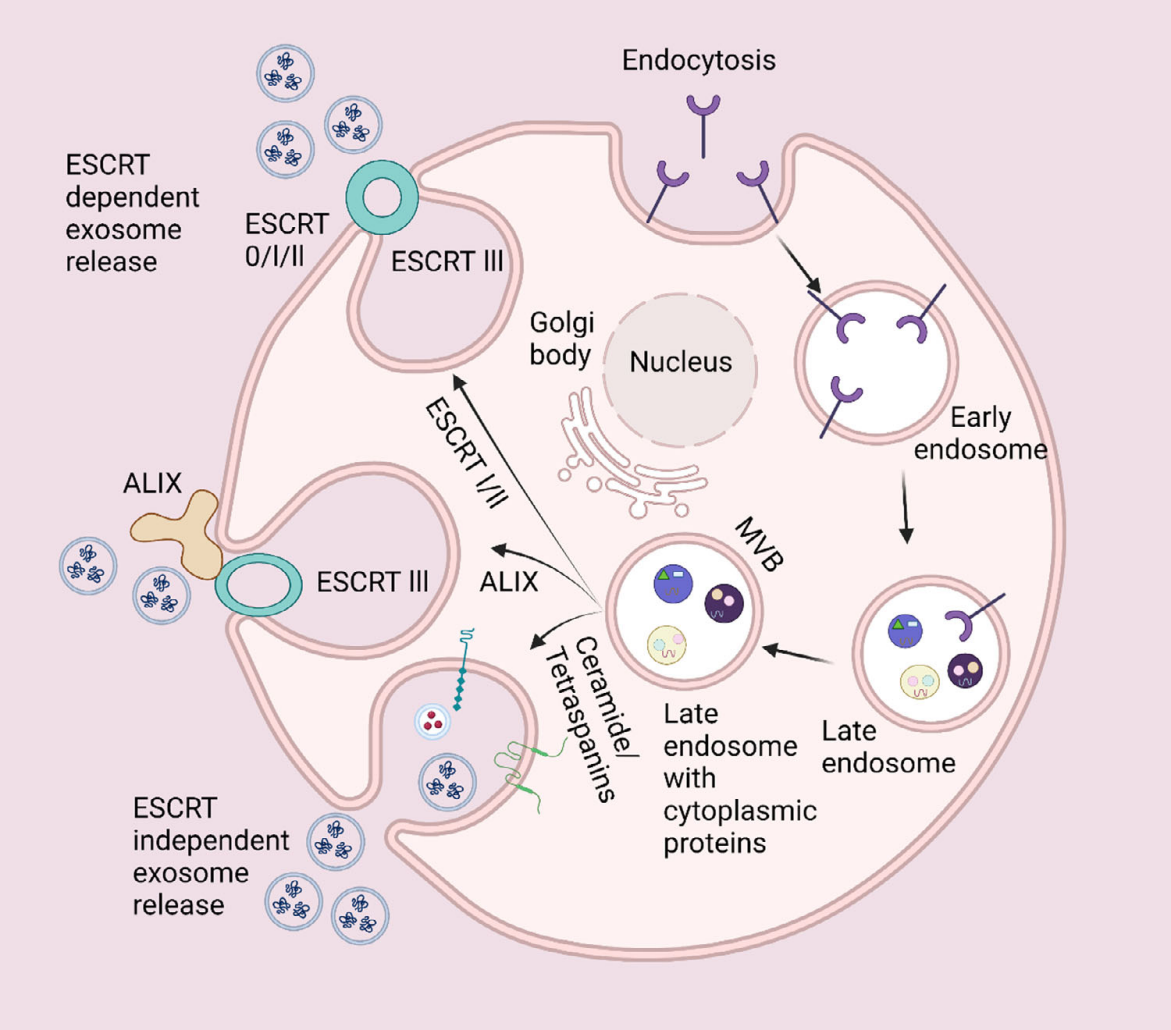
Mechanism of exosome biogenesis and release. The processes of exosomes synthesis and release is a complex series of steps that starts with the creation of ILVs inside MVBs. Through this mechanism, cells are able to carefully enclose and release certain molecules, allowing exosomes to fulfill various functions in the exchange of information between cells (created with Biorender.com).

Several crucial factors govern exosomes secretion and transport (Figure 2). Ostrowski et al. found that Rab GTPases is crucial in regulating intracellular vesicle trafficking. The Rab27 proteins, for example, regulate vesicle transport and fusion, whereas Rab35 stimulate exosomes secretion from the glial cell periphery in a GTP‐dependent manner.^[^ ^42^ ^]^ Additionally, some studies have shown that intracellular calcium ion build‐up increases exosome release, whereas low pH regulates the release of exosomes.^[^ ^43^ ^]^ Studies have also reported that diseased cell lines cultured at acidic pH promote exosomal secretion.^[^ ^44^ ^]^ Upon release, exosomal cargo can be delivered to target cells via a direct vesicle fusion with the plasma membrane, phagocytic endocytosis, or receptor‐ligand interaction. Other factors, including *N*‐ethylmaleimide‐sensitive factor attachment protein receptors, microtubule cytoskeleton, and molecular motors (dynein and kinesin), play important roles in membrane fusion, variation in MVBs distribution across the plasma membrane, and the subsequent exosome release and transport.^[^ ^1^ ^]^

## 3 CLINICAL POTENTIAL OF EXOSOMES

**FIGURE 3:**
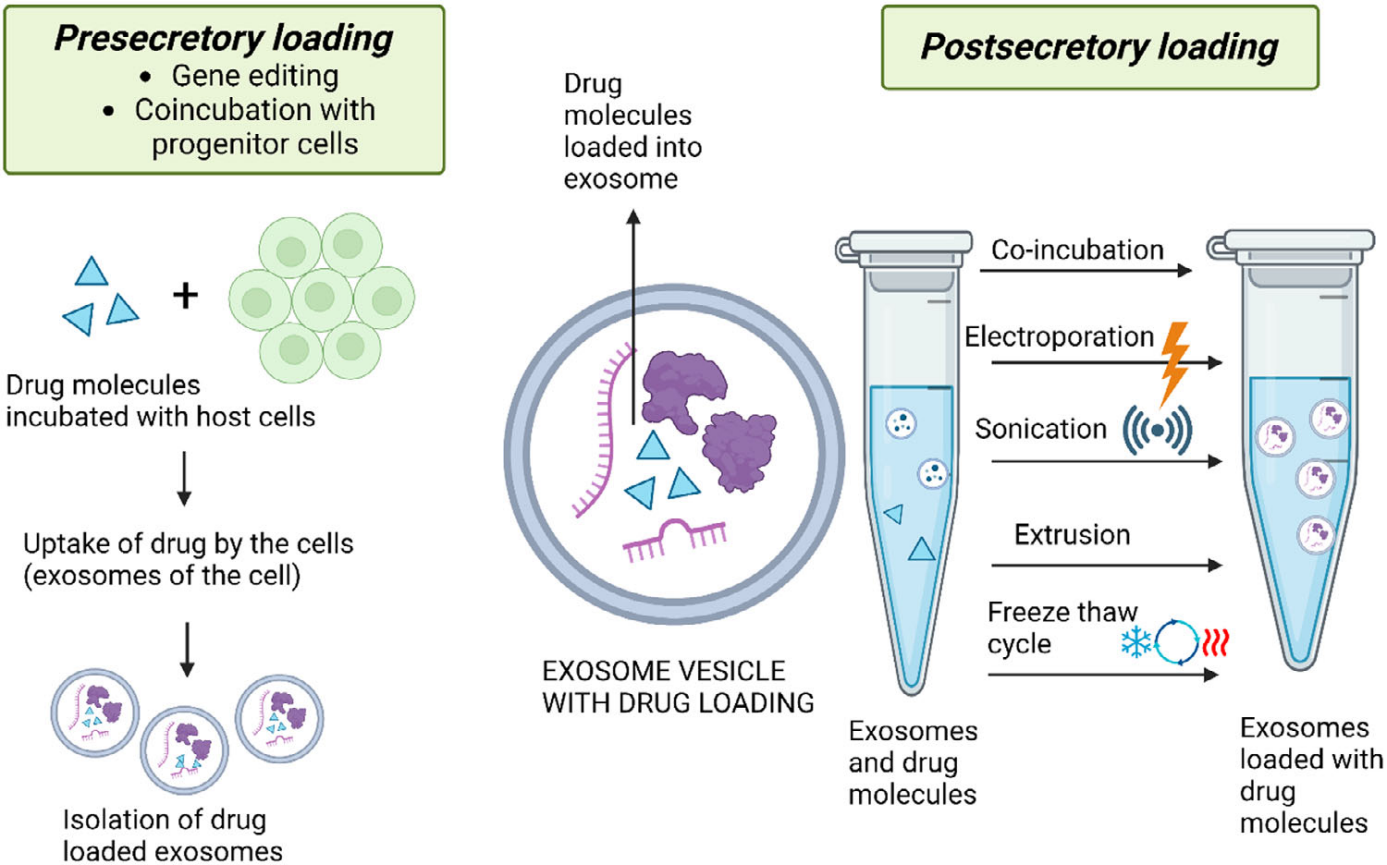
Potential of exosomes acting as drug carriers (created with Biorender.com).

Exosomes are extracellular vesicles crucial in intercellular communication. These vesicles are present in body fluids such as blood, lymph, saliva, semen, amniotic fluid, urine, ascites, and cerebrospinal fluids.^[^ ^45^ ^]^ By mimicking the origin and physiological function of the cells, exosomes contents (specific miRNAs, lipids, and proteins) can act as a biomarker or fingerprint for the donor cell.^[^ ^46^ ^]^ As a result, through their condition‐specific payloads, exosomes are a good reflection of cellular activities and can be used as biomarkers for identifying pathological conditions.^[^ ^47^ ^]^ As disease biomarkers, exosomes may provide the potential benefit of reducing and avoiding invasive examinations. Several years of research have gone into understanding the diagnostic aspect of exosomes. In 1970, microvesicles were obtained from cancer cells of a Hodgkin's disease patient. Following that, extensive research has been conducted on the potential of microvesicles as a diagnosis tool (Figure 3). Several studies have reported high microvesicles concentrations in cancer patients’ blood serum and urine. The immune‐cell‐derived exosomes also play dual roles towards cancer cells and can either activate or suppress immunological responses.^[^ ^48^ ^]^ Consequently, exosomes derived from immune cells may thus be useful in cancer diagnosis. Exosomes are currently being studied as potential biomarkers for detecting ovarian, lung, and pancreatic cancers.^[^ ^49^ ^]^

Before symptoms are observed, Parkinson's disease (PD) patients experience an earlier accumulation of α‐synuclein in the brain. Increased α‐synuclein levels are considered a PD biomarker in pathological conditions. According to the European Journal of Neurology, the amount of α‐synuclein in the brain‐derived exosomes in the blood is a promising biomarker of early‐stage PD.^[^ ^50^ ^]^ On the other hand, Alzheimer's disease (AD), as a pathological condition is characterized by the accumulation of amyloid beta plaques. Consequently, Badhwar et al. proposed that blood exosomes could be used as a potential biomarker for AD detection.^[^ ^51^ ^]^ Exosomes are gaining much more recognition as effective therapeutic agents for many illnesses because of their distinct characteristics, such as biocompatibility, capacity to regulate the immune system, and potential for precise delivery.^[^ ^52^ ^]^ Exosomes can be genetically modified to transport precise therapeutic substances, like as medications, small interfering RNAs (siRNAs), or proteins.^[^ ^53^ ^]^ These exosomes can be directed toward specific disease locations, so increasing their effectiveness in treatment while reducing unintended side effects. Exosomes produced from mesenchymal stem cells (MSCs) have exhibited anti‐inflammatory characteristics and have potential in the treatment of inflammatory conditions such arthritis, asthma, and inflammatory bowel disease.^[^ ^54^ ^]^ Exosomes may be equipped with antimicrobial peptides or antibiotics and directed toward sick cells, offering a new strategy to battle infections.^[^ ^55^ ^]^ Exosomes can be employed for targeted delivery of chemotherapeutic medicines or siRNAs to cancer cells, therefore minimizing systemic toxicity and augmenting therapeutic effectiveness.^[^ ^56^ ^]^ Exosomes can be utilized for the administration of neuroprotective substances or for the regulation of the immune system in order to address neurodegenerative conditions like AD and PD.^[^ ^57^ ^]^

Ongoing clinical trials are assessing the safety and effectiveness of medicines based on exosomes for a range of illnesses. Although the field is now in its nascent phase, the initial findings are promising and indicate that exosomes have the potential to be revolutionary medicinal agents.

### 3.1 Exosomes from tumor possess anti‐tumorigenic properties

Exosomal proteins, especially tumor‐specific antigens, are comparable to the mother cells from which they are secreted.^[^ ^58^ ^]^ Key examples are the anchored membrane glycoprotein mesothelin critical for tumor development and drug resistance, and carcinoembryonic antigens available in tumor‐derived exosomes.^[^ ^59^ ^]^ Consequently, scientists are concentrating on exploring tumor‐released exosomes for cancer vaccine development. Exosomes produced by malignant cells have been proven by research to produce anti‐tumor activity when the mother cells are genetically modified. Additionally, exosomes secreted from heat‐shocked lymphoma cells contain MHC and other co‐stimulatory molecules that trigger effective anti‐tumor T‐cell responses.^[^ ^60^ ^]^

### 3.2 Use of exosomes for regenerative medicine in orthopedics

Stem cell‐derived exosomes have demonstrated great potential as a novel cell‐free regenerative therapy. Furthermore, scientists discovered that exosomes derived from stem cells and other sources carry specific miRNAs which control cellular activities.^[^ ^61^ ^]^ Therefore, since various miRNAs play a major role in the development and treatment of musculoskeletal diseases, there is a chance for exosome‐based drugs to be used in musculoskeletal illnesses.^[^ ^62^, ^63^ ^]^ According to a research study, iRNA‐101 reduced cartilage degradation in a mono‐iodoacetate‐induced arthritic rat model.^[^ ^63^ ^]^ In a different study, miRNA‐140 and miRNA‐455 were linked with cartilage growth in bone matrix gelatin rat models, while miRNA‐9 and miRNA‐98 were associated with endochondral ossification. As a result, these miRNAs have been recognized and implicated in osteoarthritis. The conglomeration of these miRNAs in exosomes implies their therapeutic potential in osteoarthritis.^[^ ^64^ ^]^

## 4 EVS AS A THERAPEUTIC TOOL/VEHICLE IN ANTI‐INFECTIVE THERAPY

**FIGURE 4:**
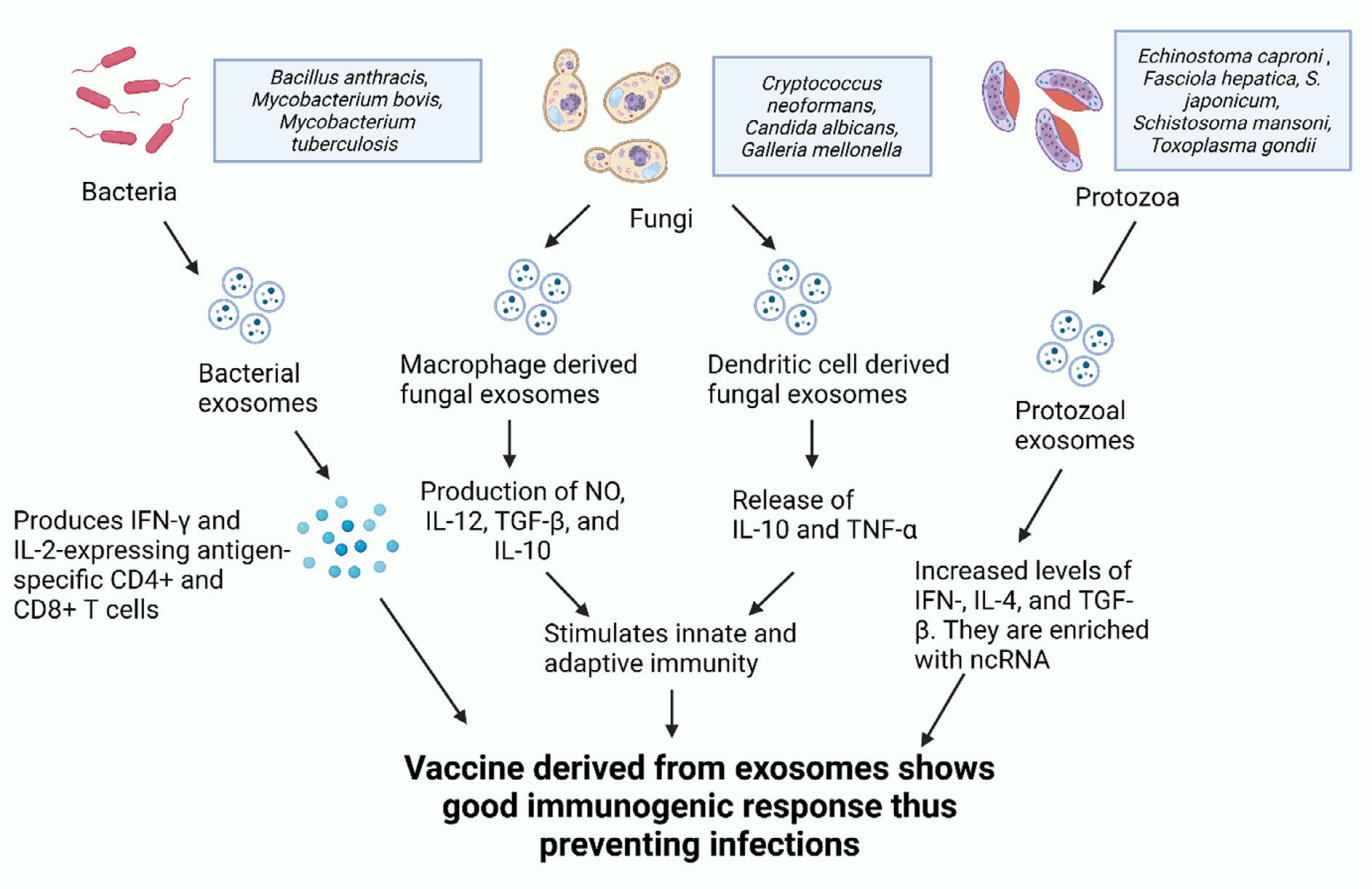
Exosome derived vaccines used for their anti‐infective potential. Exosome‐based vaccines are presently under investigation for several infectious illnesses, such as influenza, HIV, and cancer. Ongoing clinical trials are being conducted to assess the safety and effectiveness of these vaccinations, and the current findings have been promising. If these studies are successful, exosome‐derived vaccines have the potential to emerge as a novel generation of vaccinations that exhibit greater efficacy and lower reactogenicity compared to conventional vaccines (created with Biorender.com).

To eliminate the undesirable properties of medicinal compounds, researchers have recently developed synthetic drug delivery vehicles based on liposomes, cyclodextrin, modified viruses, polymeric nanoparticles, and cationic polymers.^[^ ^65^, ^66^ ^]^ However, some drawbacks, including comparatively high immunogenicity, misplaced drug aggregation (aggregation in highly vascularized organs instead of infected sites), limited systemic circulation length, and so on, are posed by phagocytic cellular uptake to these delivery systems.^[^ ^67^, ^68^ ^]^ In contrast, since they possess more favorable drug delivery system characteristics and can overcome the constraints associated with most synthetic drug delivery systems, exosomes or exosome mimetics, have emerged as a preferable alternative.^[^ ^69^, ^70^ ^]^ First, exosomes are desirable for their high biocompatibility with living tissues as they are derived from either primary human cell cultures or cell lines. Furthermore, due to the absence of exogenous factors, exosomes exhibit minimal immunogenicity and do not evoke any undesirable immunological reactions (Figure 4).^[^ ^71^ ^]^ Compared to virus‐based vectors, these vesicles exhibit a higher safety profile due to their non‐replicating nature. However, like viruses, they can selectively absorb cells through their inherent surface factors, raising the therapeutic dosage in a target tissue while lowering systemic adverse effects.^[^ ^72^ ^]^ Additionally, by improving their stability in the bloodstream, exosomes can circulate throughout the blood system under both healthy and diseased conditions. Exosomes also possess a hydrophilic center, making them ideal for delivering water‐soluble medications. Furthermore, to facilitate selective and targeted drug delivery, exosomes may penetrate biological barriers such as the blood‐brain barrier (BBB) and thereby enhance bioavailability across such barriers.^[^ ^72^, ^73^, ^74^, ^75^, ^76^ ^]^ These conclusions have been documented with support from multiple studies conducted worldwide.

There are both direct and indirect procedures for incorporating drugs into exosomes, and they include any of the following processes: electroporation (the most common method) that involves using an electrical pulse to transfect exosomes with nucleic acids; cargo loading during exosome biogenesis; and enrichment of exosomes with drugs following their exclusion and purification from recipient cells.^[^ ^16^, ^77^, ^78^ ^]^

Exosomes have also been demonstrated to trigger alterations of genetic sequences by naturally transporting nucleic acids (DNA and RNA) to specified targets (Figure 4). Given their impact on genetic sequences, exosomes have attracted considerable attention from researchers working on genetic therapy treatment strategies. Consequently, in addition to modulating gene expression, exosomes loaded with therapeutic genetic molecules have been reported to promote genetic therapies in several illnesses. Small interference RNA (siRNA), which disintegrates rapidly in the bloodstream, is used in conventional genetic therapies to interfere with the concerned genes. Therefore, by increasing the stability of these siRNAs, exosomes can aid drug delivery and facilitate the efficient transportation of siRNA to specific cells. Furthermore, exosomes can naturally transfer microRNAs (miRNAs) to the targeted cells.^[^ ^1^ ^]^ Alvarez‐Erviti et al. explored the potential pharmacological effect of exosome‐mediated rabies virus glycoprotein (RVG) peptide and siRNA delivery across the BBB in mice.^[^ ^79^ ^]^

Tang et al. discovered that exosomes enriched with HIV Tat protein could substantially stimulate primary HIV‐infected CD4^+^ T‐cells.^[^ ^80^ ^]^ In another study, microglia‐derived exosomes were used for siRNA transport across the BBB of a human telomerase reverse transcriptase immortalized human microglial cells (HTHU‐HIV) model.^[^ ^81^ ^]^ Hou et al. reported that when encapsulated with exosomes, interferon‐induced transmembrane protein 3 (IFITM3) was successfully delivered to target cells through a placental barrier to treat ZIKV infection during gestation.^[^ ^82^ ^]^ Exosomes isolated from several microorganisms, including *Trypanosoma cruzi, Plasmodium falciparum, Cryptococcus neoformans, Trichomonas vaginalis, Leishmania spp., helminthes*, and so on, have also been demonstrated to function as potential therapeutic vehicles for inflammation regulation in the human body.^[^ ^83^, ^84^ ^]^ Table 2 summarizes some of the research studies that highlighted the application of exosomes as potential drug‐delivery molecules in various infectious illnesses.

**TABLE 2:** Some applications of exosomes used as therapeutic vehicles in infections.

Exosomal cargo molecule	Method of drug loading	Change in activity due to incorporation of exosomes	Therapeutic application
HIV‐1 Tat protein^[^ ^80^ ^]^	Transfection	HIV‐1 latency reversal agent (LRA)	Anti‐retroviral treatment
Curcumin^[^ ^85^ ^]^	Mixing	Increased CD11b^+^Gr‐1^+^ cell apoptosis	Anti‐inflammatory activity
Super‐repressor IκB (srIκB)^[^ ^86^ ^]^	EXPLOR technology	Reduced proinflammatory cytokine secretion	Anti‐inflammatory activity
Transforming growth factor beta (TGF‐β)^[^ ^87^ ^]^	–	Reorganization of collagen and skin tissue development via the TGF‐β pathway	Treatment of ischemic wounds
MiR‐199a^[^ ^88^ ^]^	Transfection	Inhibition of the mTOR pathway	Improvement in chemosensitivity of hepatocellular carcinoma
MiR‐26a^[^ ^89^ ^]^	Electroporation	Inhibition of cell proliferation and migration	Anti‐tumor effect
Lysostaphin and vancomycin^[^ ^90^ ^]^	Incubation and sonication	Removal of intracellular methicillin‐resistant *Staphylococcus aureus* (MRSA)	Antibiotic effect
Interferon‐induced transmembrane protein 3 (IFITM3)^[^ ^82^ ^]^	Transfection	Suppression of Zika virus, reduction in viremia, increased delivery of IFITM3 across the placenta	ZIKV infection treatment
MiR‐138‐5p^[^ ^91^ ^]^	Transfection	Attenuation of proliferative, invasive, and migratory capabilities of bladder cancer cell lines, and reduced tumor growth	Treatment of bladder cancer
Anthocyanidins (Anthos)^[^ ^92^ ^]^	Mixing at room temperature	Increased anti‐proliferative effect	Chemoprevention of colorectal cancer

## 5 EXOSOME VESICLE AS A CARRIER FOR A VACCINE AGAINST INFECTIOUS DISEASES

Exosomes are membrane‐bound nanovesicles that can attack specific cells or tissues. By interacting with surface adhesion proteins, exosomes engage in intercellular gene exchanges and consequently alter the biological activities of the recipient cells or tissues. Moreover, based on their remarkable host biocompatibility and bio‐distribution, exosomes derived from patients’ body biofluids or organs can evade clearance by the mononuclear phagocyte system. As a result, therapeutic agents showing immunogenic responses can be incorporated inside exosomes and delivered to the targeted areas without being rapidly cleared or causing toxicity.^[^ ^93^ ^]^

Initially, exosomes were believed to be produced randomly and studies on these molecules were extremely rare. In 1983, two separate studies were performed on these molecules, attracting widespread attention from scientists. Animal model studies proved that transferrin receptors are present in reticulocyte exosomes. According to Barz et al. specific lymphomas may create exosomes with specific protein and lipid profiles that are linked to tumor immune escape and cancer invasion.^[^ ^94^ ^]^ Schirrmacher and Barz found in another study that antigens of tumor‐derived exosomes (TDEs) and their corresponding tumor cells were similar to a great extent.^[^ ^95^ ^]^ This provided a key piece of evidence demonstrating the effect of cytotoxic lymphocytes on exosome complexes and their ability to fight tumors. Raposo et al. demonstrated the role of exosomes in antigen presentation by revealing MHC‐II molecules in exosomes generated from B‐lymphocytes, which caused specific MHC‐II T cell responses.^[^ ^96^ ^]^ These findings underscored the potential of exosomes as biomarkers and a component of immunotherapeutic approaches for vaccine development.

High vaccine efficacy and uptake result in successful vaccination programs, often known as immunization. Immunization can also be defined as the process whereby a person is made resistant to disease by administering a vaccine. It involves the injection of a low‐potency or inactive pathogen into a human organ or tissue to stimulate the formation of antibodies and T cells, in addition to an immunological response that shields the person from infection. After contamination, a vaccine can potentially be used as a therapy. Historically, there have been two types of vaccines: one that contains inactivated pathogens and the other that consists of live‐attenuated pathogens. Viruses, bacteria, or other pathogenic species are used exclusively in the production of inactive vaccines. A live‐attenuated vaccine can be made in the laboratory by modifying or changing the conformation of the disease‐causing bacterium or virus.^[^ ^97^ ^]^ These immunizations can produce immunity, although they do not frequently cause sickness. Fractional vaccines can be either entirely polysaccharide‐based or largely protein‐based. The bulk of vaccines is made using polysaccharides of pure polysaccharide cell walls generated from microorganisms. Vaccines with conjugated polysaccharides include a polysaccharide chemically connected to a protein, resulting in a more potent vaccination. Subunit or subvirion merchandise, and toxoids, are included in protein‐based vaccinations (inactivated bacterial toxins).^[^ ^98^ ^]^

### 5.1 EVs for antibacterial utilization

Gram‐positive bacteria produce exosomes, which could potentially become candidates for vaccine ingredients. Rivera et al. found that a toxin released by the *Bacillus anthracis* inside the exosome vesicle produces a strong immunological response in BALB/c mice induced with the pathogen, resulting in a higher survival rate for the animals.^[^ ^99^ ^]^ Bacterial illnesses such as Tuberculosis (TB) have affected over a million people.^[^ ^100^, ^101^ ^]^ Currently, *Mycobacterium bovis* BCG is the only authorized TB vaccine, although past research indicates that it is inefficient at preventing pulmonary tuberculosis, and its protective effectiveness diminishes over time. According to the reports, various multi‐drug resistances have emerged in the current treatment of TB. Consequently, an alternative vaccine that can rapidly and safely replace or supplement BCG vaccines is required. Pramod et al. discovered 41 mycobacterial proteins in the exosomes secreted by *Mycobacterium tuberculosis*‐infected J774 cells, most of which are crucial MTB antigens.^[^ ^102^ ^]^ These discovered proteins, notably Ag85b, which was widely used in the production of TB vaccines, were demonstrated to possess excellent immunogenic properties. According to proteomic analyses, exosomes from *M. tuberculosis*‐infected and culture filtrate protein‐treated macrophages were found to contain highly antigenic proteins. Cheng and Schorey also demonstrated that when exposed to Mycobacterium tuberculosis, macrophage‐derived exosomes can produce IFN‐γ and IL‐2‐expressing antigen‐specific CD4^+^ and CD8^+^ T cells. Compared to the BCG‐vaccinated mice, mice treated with the exosome vaccine exhibited a limited TH2 response but an equivalent TH1 immunological response.^[^ ^103^ ^]^ Giri and Schorey discovered that an intranasal administration of exosomes from BCG‐infected macrophages to C57Bl/6 mice elicited a CD4^+^ and CD8^+^ memory T cell response.^[^ ^104^ ^]^ Furthermore, dendritic cell maturation and in vitro culture media activation were boosted by exosomes produced from BCG‐infected macrophages. Finally, they stated that exosomes, through their strong mechanistic property, can participate in antigen cross‐priming during a microbial attack and that exosomes with both adjuvant and antigenic properties are promising ingredients for the manufacture of TB vaccines. The Mac‐1 receptor facilitates the initiation of a modification process in neutrophils, leading to the production of antibacterial extracellular vesicles in response to *S. aureus* infection. In plants, proteins such as AGO1, RH11, and RH37 play a crucial role in loading antifungal RNA into TET8‐positive extracellular vesicles.^[^ ^105^, ^106^ ^]^ Another method in this category involves applying a layer of extracellular vesicle membrane components to nanoparticles, which has been employed in combating *S. aureus*.^[^ ^107^ ^]^ The administration of EV/ICG/MSNs combined with laser irradiation in vivo resulted in enhanced CD8^+^ T cell responses, while preserving CD4^+^ T cell responses and humoral immunity.^[^ ^108^ ^]^ Furthermore, in vivo tracking data demonstrated that the vaccine could be effectively delivered from the site of injection to the lymph nodes. EVs contain antimicrobial substances are often generated as a reaction to microbial rivals or in response to the infection of a host. Plant‐derived extracellular vesicles exert effect on the outcome of fungal and oomycete infections. Humans employ a comparable approach to manage fungal and bacterial illnesses. Microbes frequently generate extracellular vesicles to manipulate nearby organisms and obtain a competitive edge.^[^ ^109^ ^]^ Spontaneously produced extracellular vesicles from macrophages exhibited a more pronounced decrease in bacterial load compared to macrophages infected with *M. tuberculosis*.^[^ ^110^ ^]^ This indicates that the bacteria actively hinder the antibacterial action of extracellular vesicles. Outer membrane vesicles (OMVs) are naturally occurring functional nanomaterials released by Gram‐negative bacteria. They have a structure similar to cell‐derived exosomes and are known to have significant roles in several bacterial life events, including communication, biofilm formation, and pathogenicity. Recent investigations have increasingly shown the utilization of OMVs as effective antibacterial agents or carriers for delivering antibiotics. This suggests the significant potential of OMVs in antibacterial therapy.^[^ ^111^ ^]^ Chagas disease, caused by the parasite *T. Cruzi*, is another perilous illness. They infiltrate the human body by insect bites, and the mucosa membrane transports it using exosomes. The parasite is undergoing multiplication and transforming into amastigotes within the cytoplasm of the cell. Subsequently, they undergo another transformation into trypomastigotes in order to access the bloodstream by rupturing the membranes of host cells. The trypomastigotes surface is comprised of glycoprotein and TC85, which have the ability to elicit the host immunological response. The cellular reaction elicits the generation of immune cells and triggers a humoral response, resulting in the production of lytic antibodies. Both of these responses play a crucial role in regulating and combating invading infections.^[^ ^112^ ^]^ EVs play a role in immune regulation by transporting payloads, such as RNAs, from infected red blood cells (iRBCs) to immune cells. This process can either inhibit or activate the immune response, depending on the specific cellular environment.^[^ ^113^ ^]^

### 5.2 EVs for fungal infection

Fungi are a major public health concern worldwide, particularly for individuals with compromised immune systems, such as those with cancer or HIV. Invasive fungal infections have been reported to cause millions of fatalities each year.^[^ ^114^ ^]^ On the downside, drawbacks, including polyene toxicity, azole's proclivity to cause drug interactions and resistance development, and the echinocandin's narrow spectrum activity, have been associated with antifungal medications.^[^ ^115^ ^]^ Consequently, there is a need for innovative alternative preventive strategies and the Research and Development (R&D) of new antifungals to address fungal infections. Wormley et al. hypothesized in a rat lung infection model study that utilizing the IFN‐γ could stimulate immune responses against *Cryptococcus neoformans* strains. The primary infection was not only treated successfully, but the mice also exhibited total protection against a secondary pulmonary infection caused by the *Cryptococcus neoformans* strain.^[^ ^116^ ^]^ This finding, which was predicated on the use of fungi designed to produce host cytokines to trigger a protective host immunity response, represented a huge breakthrough in the vaccine's development.^[^ ^117^ ^]^ According to Vargas et al., exosomes obtained from *Candida albicans* cultures are immunologically active.^[^ ^118^ ^]^ The exposure of exosomes to dendritic cells and macrophages resulted in the production of nitric oxide and the release of IL‐12, TGF‐β, and IL‐10; and IL‐10 and TNF‐α in macrophages and dendritic cells, respectively. According to Wolf et al., the ability of exosomes derived from *Candida albicans* to activate host cells is dependent on lipid composition, as exosomes from phospholipid synthase mutations significantly prompted macrophage cytokine production.^[^ ^119^ ^]^ Exosome treatment of *Galleria mellonella* decreased the fungal burden before the development of *Candida albicans*. These findings imply that for infection prevention, exosomes derived from *Candida albicans* can potentially stimulate innate immunity and obstruct fungal pathogenesis in vivo. Furthermore, these findings support the potential applicability of fungal exosome vaccines as a viable disease prevention tool by suggesting that they stimulate host immunity via several avenues that could benefit the host during fungal infections.^[^ ^117^ ^]^ The diminished pathogenicity of the sur7Δ cells, seen in both in vitro macrophage killing tests and in in vivo insect models, may be attributed, at least partially, to EVs that do not contain pathogenesis‐related proteins. These proteins are either not synthesized by the cell or are not successfully enclosed inside the EVs.^[^ ^120^ ^]^ The EVs of *Cryptococcus neoformans* possess virulence components, including the polysaccharide glucuronoxylomannan. This polymer serves to shield the fungus from phagocytosis and impede the movement of leukocytes.^[^ ^121^ ^]^ Lopez et al. reported the presence of a 20S proteasome complex only in the EVs of the hyphal form of *C. albicans*. Given that the change from yeast to hyphae is a significant phase that increases the ability of fungi to cause disease, identifying these complexes can be used as early diagnostic indicators for invasive candidiasis.^[^ ^122^ ^]^ The ESCRT pathway specifically chooses EVs cargo that effectively regulates collective behavior during the whole process of biofilm formation.^[^ ^123^ ^]^ In general, the research on EVs in the biology of fungal pathogens and the pathophysiology of fungal infections is progressing rapidly, perhaps leading to significant changes in scientific methodologies in this subject in the near future.^[^ ^124^ ^]^ Given the effectiveness of fungal EVs in modulating the immune system, it is logical to propose that both natural and artificially created EVs hold great potential as structures for the advancement of vaccination platforms.^[^ ^125^ ^]^

### 5.3 EVs in protozoal infection

Helminths are multicellular parasitic diseases that comprise nematodes (roundworms), cestoda (tapeworms), and trematoda (flukes).^[^ ^126^ ^]^ These parasites weaken and afflict their hosts with diseases by feeding on and living inside them. When employed in the context of immune responses against infections, exosomes are known to evoke immunological reactions.^[^ ^127^ ^]^ Two trematodes, *Echinostoma caproni* (*E. caproni*) and *Fasciola hepatica* (*F. hepatica*), were particularly suggested by Marcilla et al. as capable of producing EVs that host cells can absorb, indicating the potential role these EVs play in parasites‐host communication.^[^ ^83^ ^]^ Wang et al., in their latest study on *S. japonicum* reported that mature schistosomes secrete EVs that can be absorbed by mammalian cells and that miRNA cargos control host gene expression.^[^ ^128^ ^]^ Furthermore, *Schistosoma mansoni* studies revealed that schistosomes could produce EVs enriched short non‐coding RNAs and proteins, some of which are linked to potential vaccine candidates.^[^ ^129^ ^]^ Because of recent advances in disease control, scientists are becoming increasingly convinced of the value of utilizing exosomes of antigen origin in the development of vaccines. Exosomes derived from *Toxoplasma gondii*‐infected dendritic cells (DCs, for example, were used to prevent *T. gondii* infection in mice).^[^ ^130^ ^]^ According to Johannes et al., DCs‐derived exosomes provide a defensive mechanism against Leishmania in mice.^[^ ^131^ ^]^ Furthermore, Dang et al. linked the CD63‐like tetraspanins exosome‐associated protein from the cestode *Echinococcus granulosus* to the selection of recipient cells and the use of exosomal protein‐based vaccines as a potential prevention tool against alveolar echinococcosis.^[^ ^132^ ^]^ Additionally, Maria et al. reported less severe symptoms and mortality rate, and higher IFN‐, IL‐4, and TGF‐ β levels in mice treated with isolated exosomes from *E. caproni*.^[^ ^133^ ^]^ Therefore, there is increasing evidence that exosome‐based prophylactic research may be a crucial avenue for developing innovative vaccines against parasitic illnesses.^[^ ^134^ ^]^

### 5.4 EVs in viral infection

Numerous studies have been previously conducted to determine the effects of mRNA‐based vaccinations for diseases such as influenza, human immunodeficiency virus, Zika virus, and cytomegalovirus. During the emergence of coronavirus disease 2019 (COVID‐19), the United States Food and Drug Administration (US FDA) approved mRNA‐based vaccines from Pfizer‐BioNTech and Moderna for emergency use.^[^ ^135^, ^136^, ^137^ ^]^ The market for vaccines has grown over the past four decades due to an increase in infectious disorders such as severe acute respiratory syndrome coronavirus (SARS), Ebola, hepatitis, human immunodeficiency virus (HIV), Zika, Middle East respiratory syndrome (MERS), tuberculosis (TB), severe acute respiratory syndrome coronavirus‐2 (SARS‐CoV‐2), etc.^[^ ^138^ ^]^ By the year 2024, it is anticipated that the North American vaccine market will earn approximately $24 billion. Infectious diseases such as polio and smallpox have been successfully eradicated in several countries due to ongoing vaccination programs. Therefore, the World Health Organization (WHO) launched the Expanded Program on Immunization (EPI) in 1974. EPI served as the inspiration for effective public health initiatives and comprehensive vaccination campaigns. Despite these advancements, in 2014, approximately 19 million children had not received all the doses of the diphtheria‐tetanus‐pertussis (DTP) vaccine, which is necessary for protective measures in developing countries.^[^ ^139^ ^]^ Contrarily, numerous countries have an excessive vaccination rate for children, which leaves a message that vaccination is important for public health in the current world. However, the lack of vaccine awareness remains a barrier to maximizing the rate of vaccination. Exosomes facilitate a persistent interaction between a virus and its host by triggering the activation of the innate immune system and the antiviral immune response.^[^ ^140^ ^]^ DCs release exosomes that contain functional complexes of MHC and antigen peptides. These complexes have the ability to activate cytotoxic T‐lymphocytes (CTL) in mice, leading to the development of anti‐tumor immunity.^[^ ^141^ ^]^ Exosomes originating from cells infected with HIV also carry Nef mRNA, which can be transported to human neuroblastoma cells and converted into Nef protein, resulting in the development of HIV‐related neurocognitive diseases.^[^ ^142^ ^]^ Exposing macrophages to exosomes suppressed HIV replication and triggered the activation of antiviral IFN‐stimulated genes (ISGs) and cellular HIV restriction factors (Tetherin and APOBEC3G/3F) in macrophages infected with HIV.^[^ ^143^ ^]^ In a prospective study of 24 patients with severe COVID‐19, it was discovered that a single intravenous dose of exosomes derived from bone marrow mesenchymal cells had a significant positive impact on the patients' clinical condition and oxygen levels. Additionally, this treatment led to a decrease in absolute neutrophil count and acute‐phase reactants like C‐reactive protein. Indications point to the safety and efficacy of using exosomes produced from allogeneic bone marrow mesenchymal stem cells as a potential therapeutic option for the treatment of severe COVID‐19.^[^ ^144^ ^]^

Recent developments in vaccine technology include DNA, mRNA, and protein vectors.^[^ ^145^ ^]^ Moreover, epidemiological features of numerous diseases that are prevented by vaccines have been deeply acknowledged. Collectively, these factors have profoundly altered the objectives and targets of modern immunization methods. Further, exosome‐based antiviral vaccines have grabbed the interest of researchers due to their enhanced stability, robust delivery of antigens, and potential to elicit both innate and adaptive immune responses.^[^ ^146^ ^]^ Researchers have been intrigued in employing exosomes to deliver viral antigens as they have been found to mimic the natural pathways of communication of the immune system. Furthermore, vaccines based on exosomes may be able to overcome the drawbacks of traditional immunization methods, for example, mitigating adverse drug reactions and the ability to target specific cells.^[^ ^147^, ^148^ ^]^ Macrophages that have been activated deliver miRNA29 to Huh7 cells through exosomes, which effectively inhibits the replication of HCV. The antiviral impact is nullified when either exosomes or miRNA‐29 are inhibited.^[^ ^149^ ^]^ Exosomes that carry HCV virion might potentially promote the virus and contribute to the progress of the illness, even when neutralizing antibodies are present.^[^ ^150^ ^]^ The primary oncogenic protein of Epstein‐Barr virus (a kind of gamma herpes virus) and latent membrane protein 1 (LMP1) were detected in exosomes obtained from cells infected with Epstein‐Barr virus.^[^ ^151^ ^]^

SARS‐CoV‐2 vaccination studies are underway, and they are based on the principle of neutralizing antibodies (Nabs) generation against the S protein, which prevents the binding of viral receptors.^[^ ^152^ ^]^ Research on the COVID‐19 vaccination has mainly concentrated on two parameters, that is, the level of antibodies and its potential to destroy viral particles. Numerous studies have targeted the increased production of NAb against the S protein; however, a crucial component of adaptive immunity, namely, cell‐mediated immunity, has been disregarded.^[^ ^153^ ^]^ Grifoni et al. reported the presence of SARS‐CoV‐2 CD8+ T lymphocytes that are sensitive to S and M proteins in COVID‐19 convalescent patients. In addition, Ferretti et al. confirmed the existence of the most prevalent epitopes on the open reading frame (ORF) (−1ab and −3ab), N, and M proteins. In contrast, the S protein had limited epitopes, with only one epitope on the protein's receptor‐binding domain.^[^ ^154^, ^155^ ^]^ These findings provide a better knowledge of CD8+ T cell reactivity in patients suffering from COVID‐19, along with a path to developing and manufacturing next‐generation vaccines. According to Zollner *et al*, the N protein is a strong T cell inducer.^[^ ^156^ ^]^ These findings have highlighted the relevance of CD8+ T cell reactivity in infections caused by SARS‐CoV‐2, which could be a potential vaccine target in the future. In another study, inhalable COVID‐19 vaccine comprising of a recombinant SARS‐CoV‐2 RBD coupled to lung‐derived exosomes was found to induce mucosal IgA responses, IgG antibodies, CD8^+^ and CD4^+^ T cells with Th1‐like cytokine production in the lungs of mice.^[^ ^157^ ^]^ Additionally, a separate study demonstrated that evACE2 inhibits coronaviruses that utilize the angiotensin‐converting enzyme 2 (ACE2) receptor through an antiviral mechanism. When compared to vesicle‐free recombinant human ACE2, evACE2 exhibited a potency 135 times greater in inhibiting the binding of the viral spike protein RBD.^[^ ^158^ ^]^

M and E proteins cannot exhibit adequate immunogenicity to cause antibody‐mediated responses, which is contrary to the S and N proteins of SARS‐CoV‐2.^[^ ^159^ ^]^ Studies have supported that the T cell‐mediated immune response could be initiated by exploring the sequence of more similar M and E proteins of SARS‐CoV, MERS‐CoV, and SARS‐CoV‐2, rather than the less identical S protein.^[^ ^160^, ^161^ ^]^ Numerous T cells epitope have been successfully identified in M and E proteins on SARS‐ and MERS‐CoV immunity in a previous study. These findings indicate that infections from SARS‐CoV‐2 can be prevented by utilizing these proteins as antigens for the development of vaccines. This concept can be used to obtain enhanced protection against the virus by supplying additive protection based on T cells as well as antibodies, and safeguard against SARS‐CoV variants. EV‐based vaccinations stimulate a robust immune response, leading to the development of targeted antibodies and T cells in the lungs. This immune response effectively safeguards the host from invasion by the coronavirus. Inhalable virus‐like particles with long‐term stability are believed to offer a novel approach for delivering vaccines and promoting widespread vaccination against the COVID‐19 pandemic.^[^ ^162^ ^]^

Existing vaccines of SARS‐CoV‐2 that predominantly target the S protein to generate Nabs may not provide long‐term protection. Nonetheless, T cell‐mediated immunity may alleviate this problem. Although memory B cells and antiviral antibodies are not detectable for a long time, patients suffering from these infections display virus‐specific memory CD8^+^ T cells that last 6–11 years.^[^ ^163^ ^]^ Similarly, the antibody response drops after three months in COVID‐19 patients.^[^ ^164^ ^]^ Furthermore, certain vaccinations are ineffective against the delta version of COVID‐19, which is more contagious and easily spread than other COVID‐19 variants. As a result, Nabs‐based vaccinations will not give long‐term protection against COVID‐19; amid the continuing pandemic, it is also crucial to acknowledge and consider the significance of cell‐mediated immunity for vaccine development.

Exosomes derived from living cells have become an essential tool for vaccine delivery to the targeted organ. Exosomes carry substances such as lipids, proteins, carbohydrates, and nucleic acids and contribute to cell‐to‐cell communication. They play crucial roles in physiological and pathological processes in the body, antigenic features, pathogen immune monitoring, intercellular signaling, protein and RNA production, and changing the characteristics of infectious agents. However, studies explaining the precise organic capabilities of exosomes are scanty. It has been confirmed that exosomes act as a key element in viral pathogenesis as they can deliver active molecules among different targeted cells. Li et al. verified that exosomes derived from non‐permissive liver nonparenchymal cells (LNPCs) induced antiviral properties against the hepatitis B virus (HBV) by freeing interferon‐α (IFN‐α).^[^ ^165^ ^]^ Studies have also demonstrated that exosomes transport diverse bio‐macromolecules, including proteins, lipids, viral RNAs, and regulatory RNAs. Exosomes have been found to have proteomic and lipidomic characteristics (e.g., miRNAs and small interfering RNAs). Cheng et al. found that exosomes from macrophages cause the production of a particular antigen treated with *Mycobacterium tuberculosis*.^[^ ^103^ ^]^

Exosomes‐based immunizations may contribute to the development of therapies due to their function in the development of disease, suppression of viral infection, and initiation of the host immune response.^[^ ^166^ ^]^ Exosomes and viruses are identical in terms of molecular properties such as size, content, viral production, ease of entrance into host cells, biomolecule transfer techniques, and proliferation in host cells. For example, HIV‐1 exploits the endosomal sorting complex required for transport (ESCRT) pathway to hijack exosome biogenesis to increase its dissemination into the host's body. Researchers have described exosomes and investigated their therapeutic potential in response to changes in exosomes’ contents during viral infection, such as viral particle translocation to uninfected cells and modulation of the immune response. Although classical vaccines have been used since time immemorial to prevent multiple viral disorders, they still have several drawbacks, including the possibility of virulence reversion, failure to give long‐term protection, and limited protective immunity. Currently, the improvement of the latest vaccination techniques and the shape and target of effective delivery of the drug are the main considerations.

## 6 EXOSOME VESICLES IN THERANOSTICS OF INFECTIOUS DISEASES

**FIGURE 5:**
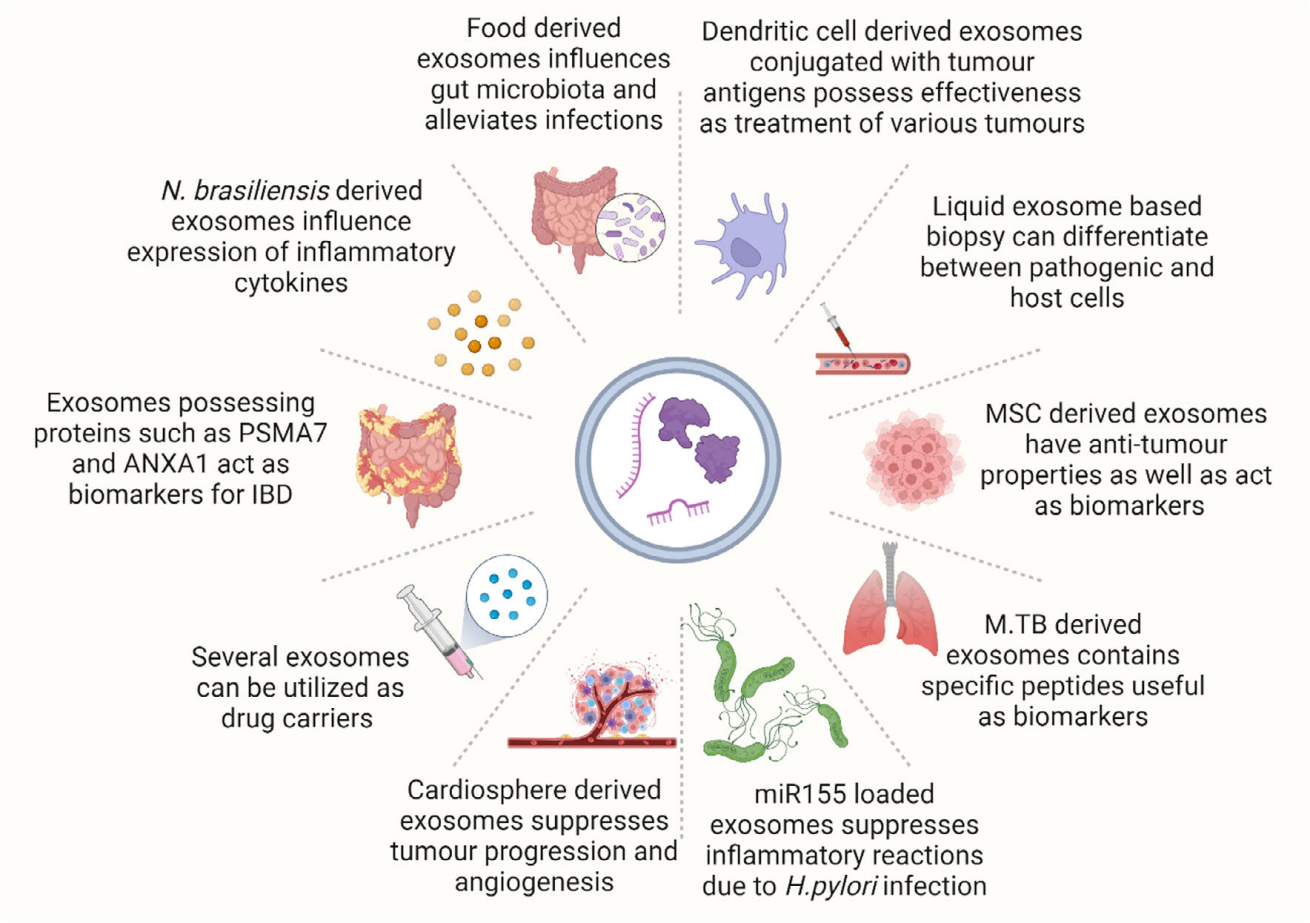
Theranostic applications of exosomes (created with Biorender.com).

Exosomes extracted from different biological fluids are implicated in various physiological functions. They are used to identify various diseases, including cancer and infectious diseases (Figure 5).^[^ ^167^ ^]^ Exosomes derived from different samples such as saliva, serum/plasma, or intestinal lumen carrying other genes/proteins could be potential biomarkers for diagnosing various infectious diseases and cancer.^[^ ^168^, ^169^ ^]^ Several studies have also reported significant variations in exosome composition (pathogen and host‐derived) between diseased and normal individuals, making them an excellent choice as biomarkers for infectious diseases.^[^ ^170^ ^]^ In confirming their biomarker potential, our previous study found that miRNA and mRNA expression patterns of circulating exosomes could serve as a diagnostic biomarker for active and latent TB‐infected patients compared with healthy individuals.^[^ ^171^, ^172^ ^]^

Mycobacterium‐derived peptides enriched within exosomes obtained across a spectrum of TB infection patients were identified and characterized using multiple reaction monitoring‐mass spectrometry (MRM‐MS) assays. This work provides a basis for exosome‐based mycobacterium‐derived peptides and can be adopted as markers for the progression of TB.^[^ ^173^ ^]^ Another study reported the expression of different profiles of exosomal miRNAs derived from tuberculous lesions, emphasizing their potential as biomarkers for the diagnosis of pleural effusion.^[^ ^174^ ^]^ *Helicobacter pylori‐*infected mice treated with microRNA‐155‐infused exosomes may have possible implications for infectious diseases. Here, miR‐155‐loaded exosomes stimulated macrophages to suppress the infection of *H*. *pylori* through modulation of the inflammatory immune response.^[^ ^175^ ^]^ Further, another study described the significant function of circulating exosomes obtained from sepsis‐related acute lung injury (ALI). It was found that selectively loaded miR‐155 collected from ALI mice promoted the growth of macrophages and inflammation by targeting specific proteins.^[^ ^176^ ^]^ Interestingly, exosomes produced by *M*. *tuberculosis* carrying mycobacterial RNA indicate that these EVs may be a good diagnostic for active TB.^[^ ^177^ ^]^ Moreover, using serum samples, a previous study found that higher levels of exosomes encoding Annexin A1 (ANXA1) induced inflammation by activating different receptors and stimulating the intestinal mucosa in patients with inflammatory bowel disease (IBD) compared with healthy individuals, which may be a promising biomarker for intestinal‐associated infectious disease.^[^ ^178^, ^179^ ^]^ The most intriguing marker appears to be mutant protein subunit alpha type 7 (PSMA‐7) derived from the saliva, which is highly expressed in IBD patients than in healthy individuals.^[^ ^180^ ^]^ Similarly, increased proteins and mRNA expression were observed in IBD patients compared with healthy individuals, aiding in IBD diagnosis.^[^ ^181^ ^]^ Collectively, these data suggest that miRNAs‐loaded exosomes are promising biomarkers and could potentially improve the clinical diagnosis of various infectious diseases.

There are numerous reports on exosome‐based‐membrane vesicles as a potential cause of biological changes. These exosome‐based vesicles purified from different types of human cells (e.g., DCs or mesenchymal cells), parasites, or plants have been found to regulate several cell types and pathways, thereby inhibiting the diseased condition.^[^ ^168^ ^]^ Although there are several limitations in developing exosome‐based therapies,^[^ ^182^ ^]^ their usage as ‘vaccines’ or ‘drug carriers’ for different diseases has risen over time. The majority of exosome‐based drug delivery studies have focused on different cancer models; however, their relevance in treating infectious conditions is less evident.^[^ ^15^ ^]^ However, reports suggest that parasite‐derived EVs could be a treatment option for IBD. An in‐vivo study reported that intraperitoneal injection of exosome‐like EVs secreted from a mouse gastrointestinal parasite *Nippostrongylus brasiliensis* produce miRNAs and protein. They also reported that EVs influenced the expression of inflammatory cytokines (pro‐and anti‐inflammatory markers) in EVs‐treated mice. This evidence suggests that EVs containing proteins and miRNAs can carry immunomodulatory efficacy and could be an option to treat infections and non‐infectious diseases.^[^ ^183^ ^]^ Moreover, food‐derived exosomal nanoparticles influence the gut microbiota and alleviate infectious‐related conditions. A study reported that plant‐based exosomal‐like nanoparticles obtained from ginger were selectively absorbed by *Lactobacillaceae* species that carry miRNAs and regulated bacterial gene expression.^[^ ^184^ ^]^ Additionally, oral administration of bovine milk‐based EVs modulates gut microbiota composition, consequently improving intestinal immunity function in mice.^[^ ^185^ ^]^ Exosomes released from *M*. *bovis*‐infected macrophagic cells may stimulate antigen‐specific cells and enhance the activation of dendritic cells. Furthermore, these may also contribute to T cell activation, thereby generating adaptive immunological responses during bacterial infection.^[^ ^104^, ^186^ ^]^

### 6.1 Engineered exosomes for anti‐infective therapy

Despite biologically‐derived exosomes having many promising applications, their therapeutic application has some limitations. Subsequently, these limitations have prompted several measures to engineer exosomes as promising nanocarriers for therapeutic application. These highly modified designer exosomes achieve the following two objectives with the help of exosome engineering: first, to enrich endogenous chemicals/drugs or biopharmaceuticals or proteins or genetic materials (mRNA, miRNA, DNA, etc.) inside the lumen or onto the exosomal surface; second, to guide/target the engineered exosomes to a specific type of cell or tissue.^[^ ^187^ ^]^

The two major exosome engineering approaches include direct or post‐isolation exosome engineering^[^ ^167^ ^]^ and parental cell‐based engineering.^[^ ^168^ ^]^ Exosomes are the precursor for engineering in the direct exosome approach, followed by exosome separation.^[^ ^187^ ^]^ Here, the cell isolation is carried out by various loading approaches such as sonication, cloaking, click chemistry, extrusion, electroporation, freeze‐thaw, incubation, and bio‐conjugation and results in the post or direct‐isolation exosome engineering approach.^[^ ^188^, ^189^, ^190^, ^191^ ^]^ Exosomes derived from recipient cells are combined with hydrophilic or hydrophobic drugs/substances before being homogenized with a homogenizer probe in the direct‐isolation sonication method, which in turn, allows the drug to penetrate the exosomes.^[^ ^192^ ^]^ The click chemistry method can conjugate substances/molecules to the exosome surface via covalent bonding. The reaction formed through direct conjugation is fast and efficient.^[^ ^190^ ^]^ The electroporation method facilitates the diffusion of drugs or substances by creating temporary pores in the lipid bilayer of the exosome membrane using an electric current. This technique enables efficient loading of therapeutic agents into the exosomes. Importantly, the stability of the exosome membrane is restored after the drug loading process, ensuring the integrity and functionality of the exosomes for subsequent applications.^[^ ^192^ ^]^ The freeze‐thaw approach allows exosomes to bind to drugs/substances at room temperature for a set period before freezing the combination in liquid nitrogen. This method is repeated for three cycles for efficient encapsulation of the drugs into the exosomes.^[^ ^193^ ^]^ Furthermore, in the direct‐isolation extrusion method, the exosomes are combined with the drugs/substances before being inserted into a syringe‐based lipid extruder.^[^ ^192^ ^]^

In the second approach of parental cell‐based engineering, precursor substances are the cells that produce exosomes, and engineering takes place before exosome extraction from cells.^[^ ^187^ ^]^ Here, different sorting modules are adopted for loading the proteins of interest inside the lumen of exosomes or onto the exosomal surface. Another method is using a short tag (WW tag) and an L‐domain motif of Nedd4 family interacting protein 1 (Ndfip1), leading to the fusion of Cre recombinase protein of interest into the exosomes.^[^ ^187^, ^194^ ^]^ The choice of exosome isolation from various sources (transfected cells, biological fluids, prokaryotic cells, eukaryotic cells, and plant products) depends on different intended applications. These exosomes are made up of a lipid bilayer with an aqueous core consisting of hydrophilic or hydrophobic drugs/substances. They may be utilized to transport RNA, DNA, and proteins as well as imaging markers for specific imaging agents, covalent linkage, or targeting ligands at the surface of the exosomes. Exosome isolation is carried out in several ways, each with its own set of benefits and drawbacks. Future studies on these engineered exosomes for prominent diagnostic and therapeutic interventions should focus on a thorough understanding of the different features and their underlying biology.^[^ ^192^, ^195^ ^]^

### 6.2 Clinical development

A study was conducted to develop exosome‐based nanovesicles loaded with an anti‐bacterial agent linezolid for use against intracellular infections of pathogenic bacteria. Here, linezolid‐loaded exosomes were engineered using a direct or post‐isolation approach by adopting the incubation technique. These exosomes exerted a bactericidal effect.^[^ ^196^ ^]^ Furthermore, there are a few ongoing/completed studies in the repository of clinical trials (clinicaltrial.gov). In one of the trials, a CD24‐carrying exosome‐based therapeutic drug has effectively completed Phase 1 studies. These engineered exosomes obtained from T‐REx‐293 cells reduce the cytokine storm infection through the overexpression of CD24 cells, which decreases the risk of clinical deterioration in patients with moderate/severe COVID‐19 disease.^[^ ^197^ ^]^ Another ongoing trial is evaluating the safety and effectiveness of MSC‐derived exosomes in moderate‐grade COVID‐19 patients with hyper‐inflammation.^[^ ^198^ ^]^ Patients are recruited for clinical trials of an MSC‐derived exosomal nebulizer against pulmonary infection.^[^ ^199^ ^]^ MSC‐EVs show great potential as a source of cell‐free treatment.^[^ ^200^ ^]^ In this study, we examined the distribution and impact of nebulized human adipose‐derived MSC‐EVs (haMSC‐EVs) in a preclinical model of lung damage. Additionally, we assessed the safety of nebulized haMSC‐EVs in healthy subjects. The study utilized DiR‐labelled haMSC‐EVs to investigate the dispersion of nebulized haMSC‐EVs in the murine model. An experimental model of lung damage in mice was created using Pseudomonas aeruginosa. The study assessed the survival rate, white blood cell counts, histology, and levels of IL‐6, TNF‐α, and IL‐10 in bronchoalveolar lavage fluid (BALF). The aim was to determine the most effective therapeutic dosage of haMSC‐EVs administered by nebulization. The MEXVT research (NCT04313647) included twenty‐four healthy participants who were administered haMSC‐EVs once, with particle doses ranging from 2 × 108 to 16 × 108. Administering nebulized haMSC‐EVs resulted in a significant increase in the survival rate to 80% after 96 h in a mouse lung damage model produced by *P. aeruginosa*. This improvement was achieved by reducing lung inflammation and histological severity. All participants exhibited good tolerance to the nebulization of haMSC‐EVs, and no significant adverse events were detected from the initiation of nebulization until the 7th day post‐nebulization. These findings indicate that administering nebulized haMSC‐EVs might be a potentially effective treatment approach, providing first evidence to support the future use of nebulized haMSC‐EVs in lung damage illnesses. In the severe stage of this illness, mechanical ventilators are employed to aid in the management of results. Nevertheless, their utilization might result in the onset of pneumonia. MSC‐derived exosomes can be utilized as an immunomodulation therapy for individuals suffering from COVID‐19 in this particular scenario.^[^ ^201^ ^]^ Exosomes have anti‐inflammatory, pro‐angiogenic, and immunomodulatory characteristics that can be investigated to enhance the outcomes of patients infected with SARS‐CoV‐2. At present, there is just one active clinical study (NCT04276987) that is explicitly investigating the therapeutic potential of MSC‐derived exosomes in the treatment of SARS‐CoV‐2‐associated pneumonia. The MEXCOVID study (NCT04276987) is a phase 2a single‐arm, open‐label interventional experiment conducted at Jinyintan Hospital in Wuhan, China. A total of seven patients, consisting of four men and three females, who were suffering from severe pneumonia due to COVID‐19, were included in the study and were administered nebulized haMSC‐Exos.^[^ ^202^ ^]^ During the nebulization of haMSC‐Exos, all patients with COVID‐19 exhibited good tolerance, without any indication of predicted negative effects or clinical instability. This was observed both during the nebulization process and immediately after. The serum lymphocyte counts of all patients showed a little rise, with a median of 1.61 × 10^9^/L compared to 1.78 × 10^9^/L. Varying levels of improvement in pulmonary lesions were detected in all patients following the inhalation of haMSC‐Exos aerosol. This improvement was particularly noticeable in four out of seven patients.

Although the number of active research involving engineered exosomes is increasing steadily, there are still numerous challenges. These challenges can be addressed with more comprehensive and systematic studies, bringing the utilization of engineered exosomes for clinical application one step closer.

## 7 CHALLENGES

Although exosomes show potential as a new and effective method for anti‐infective treatment, many obstacles impede their practical application in clinical settings.^[^ ^203^ ^]^ The variability of exosomes, arising from their varied sources and cargo content, poses a significant hurdle. The presence of heterogeneity creates challenges in establishing uniformity in the generation and characterization of exosomes, which are crucial for guaranteeing consistent therapeutic effectiveness and safety.^[^ ^204^ ^]^ Moreover, effectively directing exosomes to precise infection sites continues to be a difficulty, since exosomes may be internalized by non‐target cells or eliminated by the reticuloendothelial system prior to reaching the intended destination. Moreover, the possibility of exosomes causing an immune response, especially when they come from other species, raises worries about potential negative immune reactions. In order to facilitate the extensive clinical implementation of exosomes, it is crucial to tackle the issues of scalability and cost‐effectiveness in their production procedures. It is essential to overcome these hurdles in order to fully harness the promise of exosomes as a revolutionary treatment in combating infectious illnesses.

### 7.1 Underdeveloped methods of isolation and purification

The absence of universal standard procedures for the separation of exosome as drug carriers is a major barrier to their clinical application.^[^ ^205^, ^206^, ^207^ ^]^ As bearers of cellular information, exosomes can be found in blood,^[^ ^208^ ^]^, saliva,^[^ ^209^ ^]^ urine,^[^ ^210^ ^]^ and other biological fluids.^[^ ^211^ ^]^ Extraction and isolation of exosomes from such resources for application in drug delivery remain a daunting task.^[^ ^210^, ^212^ ^]^ Differential ultracentrifugation is a widely used separation method of exosomes. Other exosomes isolation and separation methods, including density gradients, precipitation, filtration, size exclusion chromatography (SEC), and immunoisolation have been utilized by 5%–20% of studies.^[^ ^213^ ^]^ Ultracentrifugation is a time‐consuming and labor‐intensive method. The applied G force, rotor type, rotor's k‐factor, and sample viscosity influence exosomes separation efficiency. Due to the applied external force, morphological damage and sample aggregation are observed in this method resulting in poor exosome yield.^[^ ^214^ ^]^ Whereas the ultrafiltration method is fast and isolates exosomes of high purity, the exosomes formed may be deformed or even broken due to the high shear pressures. Furthermore, the filters may get clogged, allowing polluting elements to enter the system, and interfering with downstream uses.^[^ ^214^ ^]^

Minimal information for studies of EVs 2018 (MISEV2018) defined several exosomes separation protocols based on recovery versus specificity.^[^ ^215^ ^]^ Exosomes precipitation kits/polymer (PEG), ultrafilters (Low MWCO), and ultracentrifugation methods are of high recovery and low specificity. Size‐exclusion chromatography^[^ ^216^ ^]^ and high molecular weight centrifugal filters are two approaches for intermediate recovery and specificity. SEC, immunoaffinity, and microfluidics‐based exosomes isolation methods are of low recovery and high specificity.^[^ ^215^ ^]^ Additionally, the usage of antibodies might be costly. Methods with excellent recovery and specificity are yet to be developed. Ideally, these isolation techniques should be selective, easy, economical, reproducible, high‐yield, fast, and efficient. None of the methods now in use matches these ideal characteristics. Realistically, creating consistent isolation and purification procedure will be a complex process.

### 7.2 Limited methods of characterization

Exosome separation heterogeneity results in a polydispersity of exosome sizes, making exosome characterization difficult. The size range of exosome coincide with various EVs types and cause difficulties in precise exosome isolation and identification. Spherical external membrane‐less EVs measuring 35 nm obstruct exosome separation approaches.^[^ ^217^, ^218^, ^219^ ^]^ Exosomes are polydisperse and their size range from 40 to 150 nm, making it difficult to differentiate them from other microvesicles (50–1000 nm). Furthermore, certain proteins are specific to exosome subtypes. Proteins involved in the biogenesis of multivesicular bodies, membrane fusion, and vesicle budding are more abundant in the bigger exosome subtype.

In contrast, the smaller exosome subtype possesses proteins of RNA polymerase II complex, telomere, nucleic acid, carbohydrate metabolism, and apoptosis signaling.^[^ ^217^ ^]^ Exosomal markers and proteomic analyses may be characterized incorrectly due to such protein content discrepancies. Moreover, exosome morphologies are debatable since transmission electron microscopy (TEM) morphological studies suggest cup‐shaped geometries^[^ ^220^ ^]^ while scanning electron microscopy (SEM) reveals spherical geometries.^[^ ^221^ ^]^ There is presently no direct technology for accurately quantifying and characterizing the exosomes in the pipeline. Although characterization methods such as TEM and SEM and the nanoparticle tracking system have been employed, these technologies are not appropriate for normal clinical application.^[^ ^222^ ^]^

### 7.3 Lesser drug loading capacity

The third major hurdle in using EVs for targeted therapeutics is that therapeutic payloads are loaded efficiently into EVs.^[^ ^192^ ^]^ Interested medicinal payloads can be loaded into EVs in various methods, similar to liposomes. However, EVs have a lower loading efficiency than liposomes.^[^ ^223^ ^]^ This may be due to the traces of parent cell materials in the EVs during their synthesis, leaving little room for external drug loading. As a result, loading exogenous therapeutic agents into EVs is a major issue.^[^ ^224^, ^225^, ^226^ ^]^ The sonication and extrusion method showed high catalase loading efficacy in EVs, followed by the freeze/thaw cycle and the incubation methods at room temperature.^[^ ^227^ ^]^ Similarly, the electroporation and sonication method showed high paclitaxel loading, followed by the incubation method at room temperature.^[^ ^228^ ^]^ Fuhrmann et al. observed 11‐fold high loading in the saponin and hypotonic dialysis method as that of incubation, electroporation, and extrusion methods.^[^ ^229^ ^]^ However, the incubation technique cannot load therapeutic nucleic acids, nucleic acid nanoparticles, and other hydrophilic molecules into exosomes. Loading strategies and drug hydrophobicity influence the loading capacity of EVs, with the chemical lipid composition of EVs also playing a crucial role. Furthermore, future studies should enhance present loading technologies and create novel strategies.

Electroporation—a transfection method that employs an electrical pulse to create temporary holes in the plasma membrane—drives charged molecules by producing an electric potential across the plasma membrane. Voltages for electroporation may vary from 150 to 700 V based on the type of cell. Although the mechanism is very stable and efficient, exosomes may break due to the passage of high‐voltage pulses. Because of this and the fact that membrane healing is only partial, more exosomes must be employed in this technique. Sonication is a technique that uses ultrasonic waves that increases the permeability of the plasma membrane by forming tiny and temporary holes, thereby allowing foreign material to pass through. Similar to electroporation, exosomes are damaged in this physical transfection process.^[^ ^230^ ^]^

### 7.4 Lack of effective production process

The translation of these nanosystems into clinics faces a significant hurdle in terms of finding a manufacturing approach that ensures both good quality and quantity.^[^ ^231^, ^232^, ^233^ ^]^ Researchers have endeavored to create good manufacturing practice (GMP)‐grade EVs using various approaches. Lamparski et al. proposed a GMP‐grade technique for producing, purifying, and characterizing EV‐based cancer vaccines from antigen‐presenting cells in early 2002.^[^ ^232^, ^234^ ^]^ Recently, Pachler et al. developed a GMP‐grade protocol for human mesenchymal stromal cell‐derived EVs.^[^ ^235^ ^]^ Another study reported a large‐scale method of EVs production from human cardiac progenitor cells following GMP.^[^ ^236^ ^]^ A GMP‐compliant large‐scale process of manufacturing using bioreactor for clinical‐grade exosomes has been reported. A GMP‐grade EVs manufacturing technique produces sterile exosomes with therapeutic payloads in adequate quantities for clinical testing, with no batch‐to‐batch variation that compromises effectiveness.^[^ ^237^ ^]^ Unfortunately, there is no existence of advanced technology for large‐scale production of EVs satisfying ideal GMP compliance. The critical concern for the clinical production of EVs includes scalability, repeatability, nontoxicity, potency, desired size range, surface property, and purity.^[^ ^235^ ^]^

Furthermore, the origin of the cell remains a mystery. EVs may mimic the characteristics of their native cells. The source cell type has an impact on EVs targeting efficiency and biological fate since no consensus has been achieved on the optimum EVs donor and EVs cargo. The individual EVs donor and the payload being loaded should determine the best manufacturing process for GMP‐grade EVs.

Besides these concerns, there are several barriers to employing exosomes in the delivery of drugs. Non‐specific biodistribution of exosomes into unwanted organs (such as the liver, spleen, lungs, kidneys, and pancreas) is a serious concern in exosome‐based medication delivery applications. Despite having a unique lipid and protein composition, exosomes are quickly eliminated from blood circulation after in vivo introduction. Exosomes, like PC/Chol (phosphatidylcholine/cholesterol) liposomes, remained in the bloodstream for less than 5% of the supplied quantity 3 h after injection. Exosome clearance is mostly due to macrophage capture in vivo. A previous study showed that intravenously administered B16BL6 cell‐derived exosomes were swiftly removed from hepatic and splenic macrophages.^[^ ^238^ ^]^

Furthermore, the precise process by which exosomes pass the blood‐brain barrier, which comprises astrocytes, endothelial cells, and pericytes, remains unknown. The fact that several as‐yet uncharacterized surface chemicals and cellular components of exosomes may regulate their internalization and destiny following absorption appears to be the key barrier to using exosomes for targeted treatment.^[^ ^74^ ^]^ In addition, there are no established protocols for exosome administration, such as the route (oral or injectable) and quantity of exosomes necessary to detect any effect in target cells. There is no standard unit for measuring exosomes at present. Micrograms are used in some studies to quantify exosomes; this measurement is based on determining the total protein content in exosomes, which indirectly estimates exosome quantity. Such indirect procedures cannot be used in clinical settings. Generally, exosomes are still poorly understood physiologically and biologically. The synthesis, sorting, packaging, and transportation of biomolecules, including exosomes, remain elusive.^[^ ^239^ ^]^

As mentioned above, various technical challenges must be overcome to unlock the potential of EVs‐mediated drug delivery. Therapeutics must be loaded more efficiently into vesicles, evade circulation clearance, be targeted to specific cells, and be delivered efficiently. The development of more preclinical studies in animal models would be helpful in determining which EVs would be suitable for carrying a specific therapeutic cargo to be delivered to a particular tissue. In order to overcome these challenges, a greater investment must be made in both basic research and applied research.

Having to deal with EVs in a broader sense introduces additional complexities. It is urgently necessary to standardize the isolation, quantification, and characterization of EVs. The method used for isolating EVs preparations can significantly affect their efficacy and suitability for drug loading and in vivo efficacy. When considering the imperative to scale up production, the type of EVs as a carrier becomes increasingly important. Successful translation of EVs into clinical applications depends on overcoming the major challenge of efficiently scaling up clinically effective EVs. Currently, there is no reproducible method for generating large batches of EVs from a single source, emphasizing the importance of developing sterile isolation methods with minimal impurities.^[^ ^240^ ^]^

These issues can be addressed by fostering collaboration among researchers to standardize EVs generation platforms. In the future, it may be possible to establish immortalized cell lines dedicated to generating EVs, similar to what is done in developing monoclonal antibodies. Advanced analytical methodologies, such as single‐cell RNA or genomic sequencing, are encouraged to characterize engineered EVs at the individual EVs level. Additionally, it is equally important to create an ATLAS of cells that have the capacity to generate EVs, as well as to identify the specific types of cells and tissues that are targeted by these EVs in vivo.

Furthermore, it is crucial for EVs‐based therapeutics to be approved by regulatory agencies to ensure their safety. Regulatory considerations for EVs‐based therapeutics have not been comprehensively examined compared to other nanomedicines. There is great interest in developing clinical assays and therapeutic products in EVs. A successful translation of engineered EVs into clinical applications requires bridging knowledge gaps regarding the biology of engineered EVs, as well as the resolution of engineering and analytical challenges. A commercial scale manufacturing of engineered EVs, which may be possible in the future through high‐throughput approaches such as 3D‐bioprinting, would require adequate knowledge of the necessary controls to ensure consistency. Understanding the biology of EVs biogenesis, targeting, membrane docking, and tethering in conjunction with their uptake by target cells would also facilitate the use of EVs for novel diagnostic and prognostic applications.

Although there are a number of technical challenges associated with the efficient delivery of therapeutics, the strides made over the past decade have imbued this approach with excitement and the ability to address a variety of diseases. Utilizing the innate potential of cellular EVs in the therapeutic setting aligns with the evolution of our understanding of EVs biogenesis, targeting, membrane dynamics, and uptake mechanisms. The development of new technologies, such as nanoflow cytometry and microfluidic platforms, holds promise for improving clinical outcomes and reducing healthcare costs. Despite the hurdles, the advancements in EVs‐mediated drug delivery offer a compelling and viable avenue for the future of therapeutic interventions.

## 8 CONCLUSION

EVs could be employed as drug delivery vehicles in various situations. Exosomes have a huge potential and can carry diverse synthetic and biological chemicals, which opens a new therapeutic avenue in cellular therapy. The availability of cost‐effective large‐scale production, high‐sensitivity isolation and characterization technologies to analyze batch‐to‐batch variability (and their biological repercussions) and generally applicable methods for loading drugs are all important factors in the successful translation of EVs. Exosomes as drug delivery vehicles have several advantages, including the absence of undesired exosome accumulation or absorption in the liver, as well as the avoidance of the first‐pass metabolic action before reaching target areas. Well‐characterized exosomes as well as their long‐term safety and natural capacity to transfer intercellular nucleic acids and therapeutic compounds across difficult‐to‐cross barriers such as the blood‐brain barrier would be extremely useful. The growing availability of new analytical tools is likely to reveal new insights into exosomes’ distinctiveness and may drive the development of next‐generation synthetic systems.

Subsequent research should prioritize the advancement of more effective and less harmful techniques for the extraction and refinement of EVs. This has the ability to enhance the yield and preserve the integrity of the exosomes. Enhanced characterization approaches are required to precisely distinguish and discriminate exosomes from other forms of EVs, facilitating accurate separation and identification. Research should focus on improving the medication loading efficiency of EVs. This may entail reducing the abundance of parent cell components during the synthesis process or creating innovative methods for loading. It is imperative to strive for the implementation of a manufacturing strategy that guarantees optimal quality and quantity of EVs for clinical use. This will facilitate the translation of these nanosystems into clinical settings. Research on strategies to regulate the biodistribution of exosomes might prevent their delivery to undesired organs, hence resolving the issue of non‐specific biodistribution in the use of exosomes for medicine administration. Additional investigation is required to get a more comprehensive understanding of the physiological and biological characteristics of exosomes, such as their production, sorting, packing, and transportation of biomolecules. This will provide a more profound comprehension of exosomes and their potential in pharmaceutical transportation.

## AUTHOR CONTRIBUTIONS

Vivek P. Chavda and Guanghong Luo contributed equally to this work. Vivek P. Chavda and Guanghong Luo have prepared the backbone of the manuscript. All authors have agreed with the original draft of the manuscript. Humzah Postwala and Yesha Shah have created the figures. Yanhong Duo and Vivek P. Chavda refined the first draft. Vivek P. Chavda critically revised the manuscript for intellectually correct content. All authors approved the submitted version.

## CONFLICT OF INTEREST STATEMENT

The authors declare no conflicts of interest.

## FUNDING INFORMATION

The authors received no specific funding for this work.
